# From EST to novel spider silk gene identification for production of spidroin-based biomaterials

**DOI:** 10.1038/s41598-017-13876-1

**Published:** 2017-10-17

**Authors:** Weidong Huang, Yan Zhang, Yifan Chen, Yin Wang, Wensu Yuan, Ning Zhang, Toong Jin Lam, Zhiyuan Gong, Daiwen Yang, Zhi Lin

**Affiliations:** 1 Department of Biochemistry and Molecular Biology, School of Basic Medicine, Ningxia Medical University, Yinchuan, Ningxia 750004 P.R. China; 2 School of Life Sciences, Tianjin University, Tianjin, 300072 P.R. China; 3 Department of Biological Sciences, National University of, Singapore, 117543 Singapore

## Abstract

A cDNA library from a pool of all the seven silk glands from a tropical spider species was constructed. More than 1000 expressed sequence tag (EST) clones were created. Almost 65% of the EST clones were identified and around 50% were annotated. The cellular and functional distribution of the EST clones indicated high protein synthesis activity in spider silk glands. Novel clones with repetitive amino acid sequences, which is one of the most important characteristics of spider silk genes, were isolated. One of these clones, namely TuSp2 in current research, contains two almost identical fragments with one short C-terminal domain. Reverse transcription (RT) PCR and expression analysis showed that it is expressed in the tubuliform gland and involved in eggcase silk formation. Furthermore, its single repetitive domain can be induced to form various types of materials, including macroscopic fibers, transparent film and translucent hydrogel. This study implies promising potentials for future identification of novel spidroins and development of new spidroin-based biomaterials.

## Introduction

Spider silks have been renowned for their extraordinary mechanical properties. The combination of high tensile strength and elasticity makes them comparable to the best man-made materials using modern technology^1^. In order to understand their structures-functions-property relationships, a number of spider fibroin (spidroin) cDNA/genes have been identified, including major ampullate fibroin MaSp1 and MaSp2^2–9^; minor ampullate silk gland fibroin MiSp1 and MiSp2^5,10^, flagelliform silk gland fibroin Flag^4,11^, tubuliform silk gland fibroin TuSp1^12–17^, ADF-2 (later characterized as MaSp1) and ECP or ECP-like genes^5,12,13,18^, and aciniform silk gland fibroin AcSp1^19^. Recently, silk gene from piriform (constructing attachment disc) silk gland and aggregate gland (secreting sticky glue) were also reported^20–22^.

There might be more spider silk fibroin genes awaiting isolation and characterization. In addition, exclusive of the major component from each gland, there may be other minor fibroin components which also play roles in silk fiber formation. For example, in addition to the major component of TuSp1, ADF-2 and ECP-1,2 were isolated from the tubuliform gland or egg case^5,12,13^, which may be involved in the silk formation and/or conformation transition of the silk fiber assembly.

Besides the attempt of identifying novel spider silk cDNA/genes, many efforts have been put on the spinning process, through which the soluble highly concentrated fibroin dope is converted to insoluble fibers^23–27^. It is indicated that spinning conditions play a role on the mechanical properties of the fibers^26^. In this spinning process, pH decreases^28^ and ion content changes^26,29^ along the spinning duct. At the same time, water content decreases drastically in the concentrated protein solution. Although some models were suggested for the spinning process, molecular information for elucidating this sophisticated process is still incomplete. Therefore, it is necessary to identify the molecular basis which underlies the complex molecular events of silk formation.

Sequencing of randomly selected cDNA clones (EST, expressed sequence tag)^30^ has been proven to be a relatively inexpensive and efficient means to rapidly access many of the expressed genes of a specific tissue or organism. This method is important for providing the expression profile of the source tissue. It has also been commonly used to identify novel genes involved in specific biological processes, and is especially valuable for organisms where genomic data are limited.

In this study, we used the EST method to identify the molecules involved in spider silk formation. Mainly because of its availability and large body size, a local species of the orb-web spider, *Nephila antipodiana (N*.*a*.*)*, was used in this study. The expression profile of spider silk glands was analyzed through our EST data and a number of molecules that have implications in silk fibroin synthesis, silk formation or procession were identified. We also report the identification of a partial novel cDNA molecule, which is specifically expressed in the tubuliform gland of *N*.*a*. and involved in eggcase silk formation.

## Results and Discussion

### Overview of EST clones in spider silk glands

A cDNA library from the 7 types of silk glands of 5 adult female *N*.*a*. was constructed. The titer of the primary library was 7.5 × 10^6^ pfu while that of the amplified library reached 9 × 10^11^ pfu/ml. The empty clone percentage was 1.8% and the average insert length was approximately 900 bp, which ranges from 300 bp to 3000 bp. These data are summarized in Table 1. Following the method of size selection and sequencing, 1076 randomly selected clones from the spider silk gland cDNA library were single-pass sequenced from the 5′ ends. Fasta analysis showed that 698 of the 1076 clones had statistically significant similarity (Poisson P value ≤ 10^−5^, sequence overlap > 150 bp) to the available sequences in the public DNA database. This high identification rate may be mainly due to the pre-selection of the insert size, leading to sequencing only the coding region, which is more conservative than the 3′ UTR (un-translated region). Besides, more and more DNA sequences are available, providing a larger pool to compare with. Sequences of 505 EST clones (with informative functions) generated from this study have since been submitted to NCBI dbEST (NCBI access no. GT029466 to GT029977). The remaining 378 clones did not share significant sequence homology with any known sequences in the databases; and are under further investigations (Table 2).

**Table 1:** Summary of cDNA library.

cDNA Library	Statistical data
Size of primary library (pfu)	7.5 × 10^6^
Titer of amplified library (pfu/ml)	9.0 × 10^11^
Range of insert length (bp)	300–3000
Average insert length (bp)	900
Empty vector clone (%)	1.8

**Table 2:** Summary of spider silk glands EST clones^1^.

	Number of clones	Percentage
Sequenced	1076	100%
Identified	698	65%
Annotated	511	47%
Non-redundant	240	22%
Redundant	271	25%
Full length	87	8%
No match	378	35%

^1^Classification of 1076 EST clones based on their characterization study. Sequenced: a clone was sequenced and filed in this study; Identified: a clone matches a sequence at the amino acid level in the public database of Fasta3; Annotated: a clone is similar in sequence to a protein or DNA whose annotation is available in the database; Non-redundant**:** an annotated clone that appeared only once in EST library; Redundant**:** an annotated clone that appeared more than one time in the EST library; Novel: a clone that was isolated and reported for the first time in spiders in this study; Full length: a clone contains deduced complete protein coding regions; No match**:** a clone that shares no significant sequence homology with any sequences in the Fasta3 database.

### Distribution of the identified clones in spider silk glands cDNA library

As cellular localization of a protein is closely related to their biological functions, the identified EST clones from the spider silk glands (698 clones from the 7 types of silk glands) are classified into seven broad categories largely according to the major cellular localization of their encoded proteins: cytosolic proteins (CS), cytoskeletal protein (CK), membrane proteins(MP), nuclear proteins(NP), secreted and extracellular proteins(SP), translational machinery proteins(TM), mitochondria proteins (MT) (including both nucleus coded and mitochondria coded proteins) and the clones encode proteins with unknown location due to the shortage of information (UN). The distribution of cellular localization of the EST clones is listed in Table 3. The cytosolic proteins (30%) and proteins involved in translational machinery (22%) exceeded 20%. As we know, the cellular distribution of the genes expressed in one tissue represents roughly the energy distribution of this source tissue. It thus provides us a profile of the energy flow and the molecular events involved in it. Since the spider silk glands are highly active in silk protein synthesis, it is logical to have a high percentage of translational machinery molecules. In the cytosolic protein group, many of them are involved in protein folding and sorting, indicating the vigorous activities of post translational modifications and strict controls for the fibroin synthesis in the silk glands. The category of unknown EST clones included 31%, which may be due to the fact of limited molecular information from the silk glands (65%). The functional information (unknown category) is not available at current stage. There might be novel cDNA molecules involving specific biological functions in the spider silk synthesis included in this category, which needs to be further studied.

**Table 3:** Distribution of identified EST clones in the cDNA library^1^.

Category	Clones	%
CK	13	2
CS	208	30
MP	35	5
MT	27	3
NP	21	3
SP	23	4
TM	153	22
UN	218	31

^1^The percentages of identified clones for different categories in the cDNA library are show. TM, translation machinery; CK, cytoskeleton; MT, mitochondria; CS, cytosolic; MP, membrane proteins; NP, nuclear proteins; SP, secreted and extracellular proteins.

### Functional distribution of EST clones

Since it is not practical and not necessary for us to discuss all the genes identified with only statistical data, we try to focus here some interesting phenomena acquired from the EST pool. Based on the occurring frequency of the cDNA clones, we listed the most abundant cDNA clones from the 7 types silk gland library (Table S1, clones appear for at least 3 times, separated in three parts, which include clones of translational machinery; general clones, and clones of heat shock proteins). There include 223 clones, representing 21% of the clones (223 clones/1076 clones) from the library. In this list, some of the clones represent house-keeping genes or the genes involved in mainstream metabolism, such as ADP/ATP translocase, actin, glyceraldehyde-3-phosphate dehydrogenase, cytochrome oxidase subunit I, aldehyde dehydrogenase, carboxylesterase precursor, techylectin 5 A, IgE-dependent histamine release factor, and ubiquitin; and the rest may play more specific roles in the silk protein synthesis. In addition, 3 cDNA clones similar to excretory/secretory mucin MUC-5 gene (muc-5) (*Toxocara canis*) are also identified from *N*.*a*. silk glands. The MUC-5 gene was isolated from the mucin coating the *T*. *canis* labile surface^31^. It is a secreted glycoprotein and the exact function of this gene is not clear. However, the abundance of this cDNA clone suggests that it might play a role in spidroin synthesis and silk formation, for which we try to provide more detailed discussions.

### Silk genes

Seven clones of known silk genes, representing major ampullate silk fibroin gene *MaSp1* (4 clones), minor ampullate silk gene *MiSp1* (1 clone)^32^, tubliform silk gene *TuSp1* ^14^, and aciniform silk gene-like are identified^33,34^. So far, there are a number of silk genes reported from spiders and our ESTs sequencing results, consistent with the sequences of previously identified silk genes, demonstrated that current strategy is effective in silk gene isolation.

Recently, large scale transcriptome analysis methods have been applied to identify new silk genes^18^. ECP homologues in an araneid was thus found. Newly developing methods are automatic and time-saving. However, due to the extremely long and highly repetitive nature of silk structural genes, there might be technical limitations to annotate the transcripts efficiently.

### Genes involved in spidroin synthesis and storage

As shown in Table S1, a large number of RNA genes and translational elongation factors (12% or 132/1076 clones) are present in our EST data. The most abundant elongation factor is the elongation factor 1 alpha, which binds to aminoacyl-tRNA during translation elongation. This is quite reasonable since silk proteins are synthesized at an extraordinary rate in silk glands. Four copies of sec. 61 alpha subunit and three copies of ubiquitin cDNA clones are present in the EST data. Sec. 61 alpha subunit is a major component of the Sec. 61 complex, which forms the protein-conducting aqueous channels in the ER membranes^35^. This result may indicate that silk protein synthesis in silk glands is under the ER quality control, to ensure that the silk proteins are correctly folded for silk spinning. In addition to Sec. 61 and ubiquitin, a number of heat shock proteins, including Hsp70, Hsp60, Hsp20.8 A and small heat shock/alpha-crystallin protein precursor, are found in the silk gland (Table S1). They are the major chaperone molecules and are believed to prevent aggregation of unfolded proteins and to facilitate nascent proteins for their folding and assembly^36–38^. The identification of various heat shock proteins from spider silk glands may therefore provide clues for understanding of the process involved in fiber silk protein folding and assembly and quality control. Ornithine decarboxylase (ODC) is found to be the most abundant gene expressed in spider silk glands. 70 cDNA clones encode for ornithine decarboxylase, accounting for 6.5% of the total 1076 identified cDNA clones. ODC is the rate-limiting enzyme of the polyamine synthesis and involved in diverse biological processes, including cell growth, differentiation, transformation and apoptosis^39^. In silk gland, however, the functions of ODC are yet to be elucidated.

### Novel cDNA clones containing repetitive sequence

**Figure 1:**
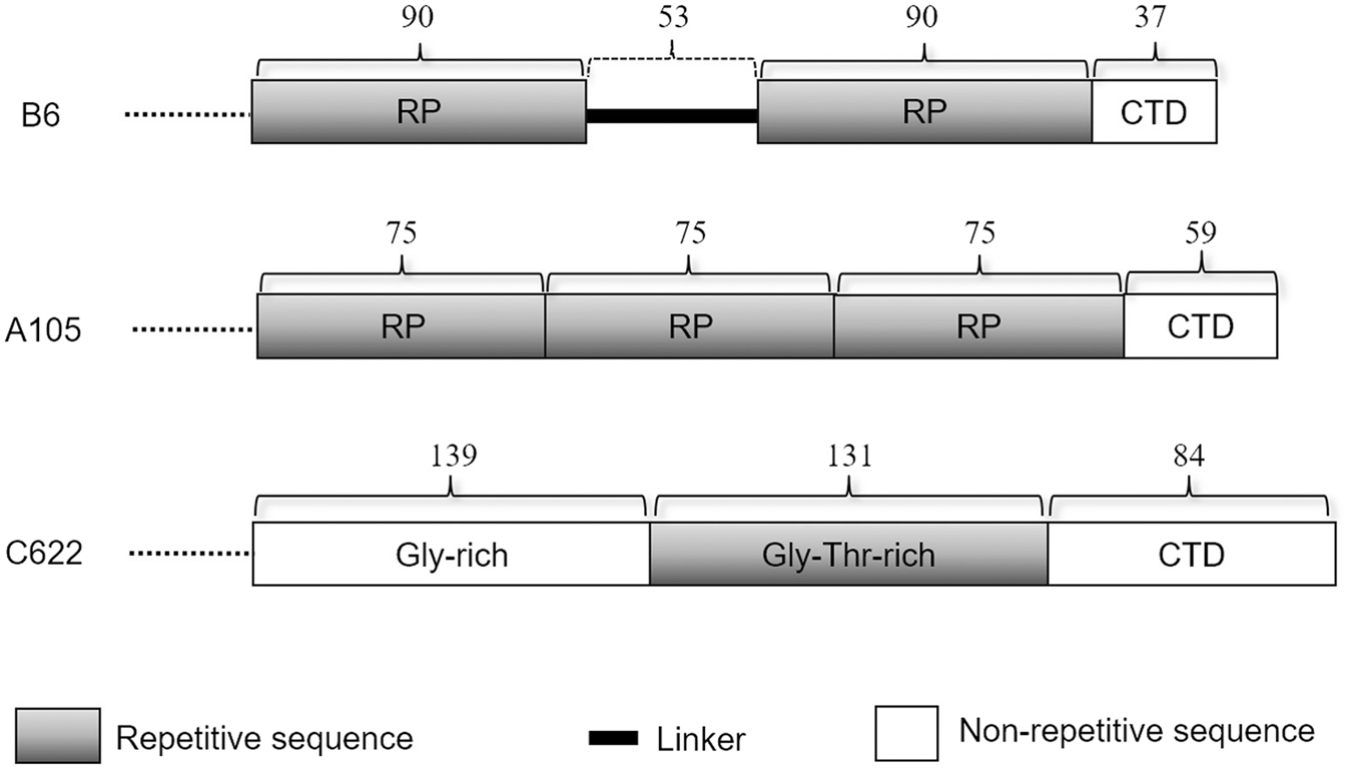
Architectures of representative clones with repetitive domains or motifs. All clones contain partial sequences. Repetitive and non-repetitive domains are represented by bars. The amino acid number of each domain is indicated above the corresponding bar. RP: repetitive domain; CTD: C-terminal domain.

**Figure 2:**
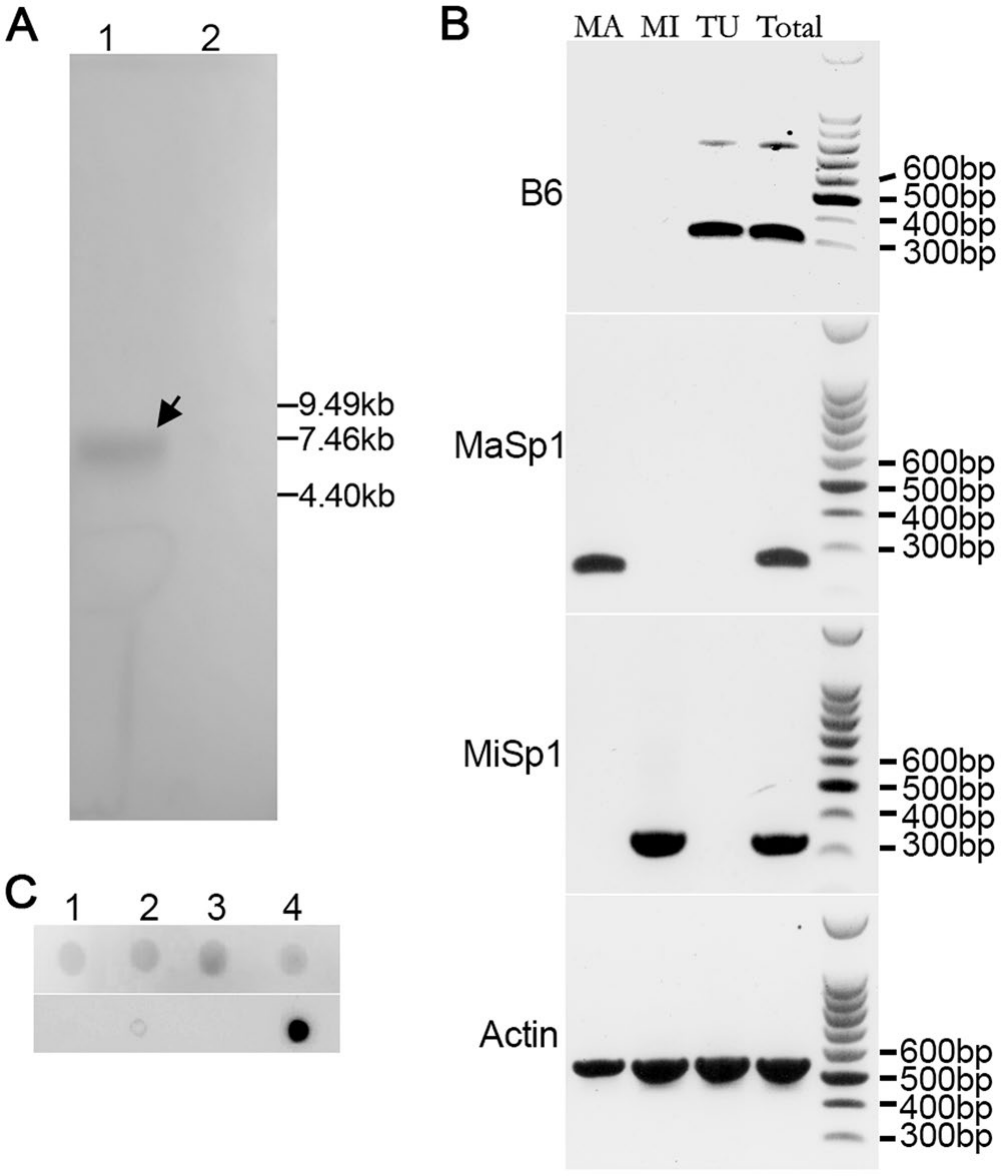
Expression and assembly of TuSp2. A. Full-length northern blot analysis showing the expression of TuSp2 in *N*. *a*. spider. Lane 1: total RNA sample from silk glands of *N*.*a*. was loaded and separated on a 1.0% agarose-formaldehyde gel, transferred to nylon membrane, and probed by *TuSp2* probes. The hybridized band is approximately 8 kb as indicated by an arrow. The small circle below the hybridized band is a pencil mark and consistent to the rRNA position. Lane 2: total RNA from non-silk gland tissues as a negative control. B. Full-length gel profiles of RT-PCR to analyze the mRNA level of EST clone B6. Total RNA from major ampullate silk gland (MA), minor ampullate silk gland (MI), tubuliform silk gland (TU) and mixture of seven types of silk glands (Total) were isolated and used to synthesize cDNA. RT-PCR primers specific for actin, MaSp1, MiSp1 and B6 were applied and the PCR products were analyzed. C. Full-length figure of dot blot showing involvement of TuSp2 in eggcase silk formation. Upper: protein staining; lower: dot blotting. Lane 1: dragline silk; Lane 2: eggcase silk; Lane 3: total silks without egg cases; Lane 4: recombinant TuSp2-RP.

In present study, 378 EST clones (38%) showed no significant similarity to those in the public database and 219 contain deduced animo acid sequences with 107 of them contain deduced amino acid sequences without any stop codon in the middle. To find out repetitive motifs, the software Vector NTI was used to translate cDNA sequences manually into different reading frames with online statistical resource (http://www.ebi.ac.uk/Tools/saps/). As a result, more than 10 of the 107 deduced protein sequences were found to contain repetitive sequences at the amino acid sequence level, which is one of the most important features of spidroin genes. Schematic figure representing the comparative organizations of three representative EST clones is showed in Fig. 1 while their sequences will be available upon request (B6 with NCBI accession no. EF044306., while the others are still under investigations). Both clones B6 and A105 contain multiple repetitive domains (RP) and C-terminal non-repetitive domains (CTD), while clone C622 contains one glycine-rich (15%) and one glycine-threonine-rich (57%) domains with a longer C-terminus. Short repetitive motifs, such as GT(S)XTGT(S)T(S), are present in glycine-threonine-rich domain in C622. We now designated B6 as gene *TuSp*2 (tubuliform spider fibroin 2), following the convention of *TuSp1* since they are both specifically expressed in the tubuliform gland and involved in eggcase silk formation (Fig. 2, see below)^14^.

### Sequence and size of gene *TuSp2*

The EST clone of *TuSp2* encodes a partial cDNA of 1,194 bp, with an open reading frame of 270 amino acid residues (Fig. S1). It contains two almost identical fragments: one from amino acid residue 1 to 90 and the other from 144–233, which is rich in serine, alanine, asparagine and leucine residues (Fig. S2). There is a difference of 18 nucleotides in these two fragments, leading to 12 different amino acid residues. The organization of this molecule is shown in Fig. 1. The linker between residue 91 and 142 could also be part of RP domain. In order to validate the existence of this gene, Northern blot analysis was applied and found that the full-length *TuSp2* reaches approximately 8 kb in length (Fig. 2A). This result indicates that *TuSp2* likely encodes a fibroin protein with high molecular weight of over 200 kDa. Till now, mainly two types of eggcase silk genes: the huge *TuSp1* (or *CySp1*,*2*) and the much smaller *ECP* or ECP-like genes have been identified in tubuliform gland^14,17,18^. The repetitive domain of TuSp2 shows low sequence identity ( < 25%) to those of TuSp1, MiSp1, AcSp1 and MaSp1 (Fig. S3). TuSp2 consists of an unusual short nonrepetitive C-terminal domain of 37 amino acid residues and shows no similarity to those of any known spidroins.

Very recently, the first genome of orb-weaving spider *Nephila clavipes* (*N*.*c*.) was uncovered^40^. Genome BLAST analysis revealed a partial sequence with ~60% identity to TuSp2 in the amino acid level (Fig. S4). This partial sequence in the *N*.*c*. genome involves one full RP domain and a short C-terminal domain of ~55 amino acid residues, suggesting that TuSp2 could also exist in *N*.*c*. silk gland. However, other RP and the N-terminal domains of putative TuSp2 from *N*.*c*. are missing, most likely due to the incompleteness of the *N*.*c*. genome.

### Transcriptional distribution of gene *TuSp2*

A number of RT-PCR primers were designed to apply RT-PCR reactions to identify the expression pattern of *TuSp2*. As shown in Fig. 2B, TuSp2 mRNA was detected solely in the tubuliform gland and the pooled silk gland. As controls, MaSp1 and MiSp1 mRNAs were detected in the major and minor ampullate glands respectively, as well as in the pooled silk glands, but not in the tubuliform gland. Furthermore, ubiquitously expressed actin mRNA was detected in all four samples (Fig. 2B). Under current experimental condition, the RT-PCR reactions were limited to less than twenty-five cycles, and the results indicated the priority of individual genes in various glands. To further investigate the properties of *TuSp2*, a miniature construct of single repetitive domain of TuSp2 (TuSp2-RP) was expressed as a soluble recombinant protein in *E*. *coli*. Purified TuSp2-RP (Fig. S5) was applied as antigen to raise multi-clonal antibodies. Dragline, eggcase and total silks without egg cases were collected, dissolved and blotted onto the PVDF membrane. As shown in Fig. 2C, only eggcase silk demonstrates clear cross reactions to the antisera against recombinant TuSp2-RP. It was thus confirmed that TuSp2 is specifically assembled into eggcase silk fibers.

### Spidroin-based biomaterials from TuSp2

**Figure 3:**
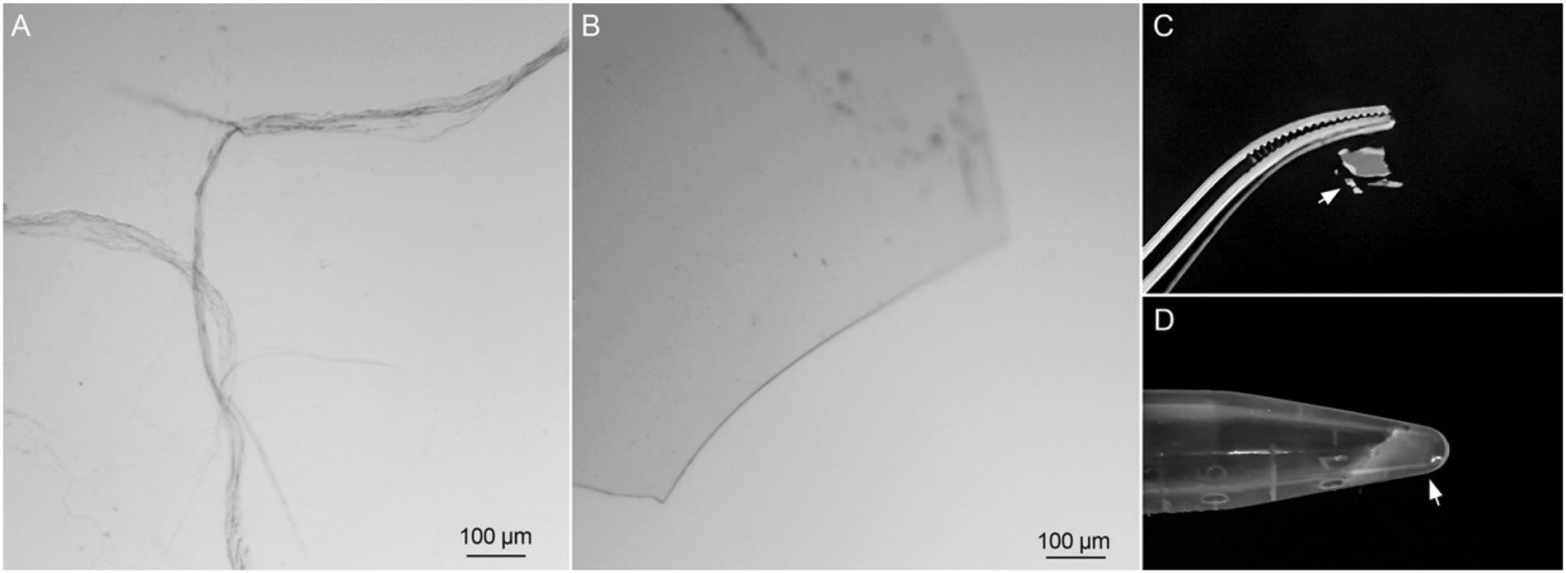
Spidroin-based biomaterials formed from TuSp2-RP. (**A**) Appearances of artificial fibers, scale bar: 100 µm. (**B**) and (**C**) Transparent film (indicated by an arrow), scale bar: 100 µm. (**D)** Translucent hydrogel (indicated by an arrow).

It’s well known that silk fibroins can be processed into many material forms, including fiber, film or hydrogel. We found that single TuSp2-RP in water solution at a concentration of at least 10 mg/ml can be transformed into macroscopic fibers upon physical shearing at room temperature (Fig. 3A). Since the fibers tend to stick to each other, it is unlikely to acquire a single string of fiber for mechanical tests. TuSp2-RP can also be induced to form a transparent film structure upon drying on a petri dish or self-assemble into translucent hydrogel material at a pH close to its pI (Fig. 3B–D) at room temperature. Due to the low molecular weight of TuSp2 single domain (~15 kDa), the transparent film and translucent gel are too brittle to investigate their physical properties. Nevertheless, the ability of TuSp2 domain to form various structures provides promising potentials for the development of high-molecular-weight TuSp2 with multiple RP domains for biomaterial applications.

In summary, we have constructed a cDNA library from a pool of all the seven silk glands and found out a number of EST clones containing novel repetitive amino acid sequences, which possess no significant similarity to those of known silk proteins. On the basis of both sequence and biochemical data, we infer that *TuSp2*, different from both *TuSp1* and *ECP* genes, is a novel gene involved in eggcase silk formation. We also demonstrated the capability of TuSp2 repetitive domain to form various types of materials. Current study implies promising potentials for identification of novel spidroins and development of new spidroin-based biomaterials in the future.

## Methods

### Sample collection and construction of a silk gland cDNA library

Five adult female spiders of *N*. *antipodiana* were collected from the bush in eastern part of Singapore. In order to collect spiders with active silk biosynthesis, a large portion of the spider web was destroyed one day before collection. Only spiders found to repair their webs were collected. Tissues comprising of the seven types of silk glands were isolated and total RNA was extracted using Trizol reagent (Gibco BRL). Poly-A RNA was purified using an Oligo-dT affinity column (Stratagene). For cDNA library construction, Stratagene’s Lambda Uni-Zap XR cloning system was used according to manufacturer’s protocol. In brief, ~5 µg of Poly-A RNA was extracted and used for cDNA synthesis. Two restriction enzymatic digestion sites of XhoI and EcoRI were introduced. cDNA molecules were directionally cloned into the predigested Zap vector and packaged using the package extract according to manufacturer’s instruction manual (Stratagene). A total of 7.5 × 10^6^ pfu (plaque forming unit) of bacteriophage were obtained in the original library and subsequently were amplified using thirty 150-mm culture dishes. The titer of the amplified library was 9 × 10^11^ pfu/ml and the amplified spider silk gland DNA library was then aliquoted into 60 Eppendorf tubes and stored at −80 °C for long term storage. The percentage of empty clones determined using the blue-white selection by PCR amplification with T3 and T7 primers.

### cDNA clone selection and sequencing

The phage library was converted to the plasmid library by mass *in-vivo* excision according to the standard protocol (Stratagene). To improve the possibility of accession the coding region, PCRs were performed using T3 and T7 primers for selection of cDNA clones with large inserts ( >1 kb) for sequencing reaction. DNA sequencing was carried out using the ABI BigDye Terminator (Version 3) and the ABI PRISM® 3100 sequencer (Applied Biosytems). Each clone was sequenced from the 5′ end using the vector primer T3 and sequencing readouts > 600 bp were usually obtained.

### Sequence homology search and analysis

After removal of vector sequences and correction of obvious reading errors, all EST sequences were submitted to Mail-Fasta via email (fasta@ebi.ac.uk) for sequence homology search. The search was against all the DNA entries in the EMBL database (http://www.ebi.ac.uk/Tools/fasta33/index.html, UniProt). The first 100 most homologous sequences were listed, and usually the best 10–30 sequence alignments were examined. The identification of a spider homologous cDNA clone was based on the smallest Poisson probability, P(N), reported by the mail-Fasta analysis. In general, the highest P(N) value accepted for clone identification was 1 × 10^−5^. An identified clone generally shares a high sequence identity (usually > 65%) over a relatively long fragment ( >150 bp) with the most similar sequence homolog. We mainly searched through literature to decide the cellular localization of the characterized EST clones. The clones not sharing reliable similarities to known sequences in the database were classified in the category of ‘unknown’. In particular, nucleotide sequence of the EST clone B6 reported in this paper belongs to this ‘unknown’ category and was submitted to the Gene Bank database under the accession no. EF044306.

### Northern blot analysis

Total RNA from the spider silk glands (~1–10 μg) was separated on a 1.0% agarose-formaldehyde gel and blotted onto a nylon membrane (Roche) with 10 × SSC (1 × SSC is 15 mM sodium citrate, 150 mM NaCl), crosslinked by an automatic crosslinker (Stratagene). The cDNA fragments from EST clone of were labeled with Dig DNA labeling kit (Roche). Hybridization was performed and was followed by immunological development according to the manufacturer’s instructions by chemiluminescent method using CSPD substrate (DIG Luminescent Detection Kit, Roche, Switzerland).

### RT-PCR analysis

Total RNA from major ampullate gland sac, minor ampullate sac, tubliform gland and mixed tissue of all the seven types of silk glands was isolated with Trizol (Invitrogene). First strand cDNA was synthesized from the total RNA with SuperScript RT (Invitrogen). PCRs were carried out using Taq polymerase (Promega) with 25 cycles. The primers used for PCRs are:

B434-RT-F (5′-TTGATCTTGCTGGACGGGACTTGA-3′) and B434-RT-R (5′-TCCACATCTGTTGGAAGGTGGACA-3′) for actin;

D938-RT-F (5′-CTTCACGCTTATCTTCTCCTGAAG-3′) and D938-RT-R (5′-AACAATCTGAGCAGCTTGACCAG-3′) for MaSp2;

145-RT-F (5′-CTGGAAGTGCTGCAGGAAATGCTT-3′) and 145-RT-R (5′-ACTAGAAGCAGCGGAAGATGCAGT-3′) for MiSp1;

B6-RT-F (5′-CAGGACCAAACTTGTCTATTGGAG-3′) and B6-RT-R (5′-GAGGTTTACCATCGCTGAAGAATG-3′) for clone B6.

### Construction, expression and purification of TuSp2-RP

The cDNA encoding TuSp2-RP (90 amino acid residues) with a linker at its N-terminus (52 amino acid residues excluding the first proline residue) was amplified from B6 cDNA using one pair of primers:

5′-CGCGGATCCAACCTGAGCATTGGCGATACC-3′

5′-AATCTCGAGTTAGCCAATTTCCACCATTTTTTT-3′

The PCR product was subcloned into a pET-M expression vector between BamH I and Xho I restriction sites. pET-M was derived from pET-32a (+) expression vector (Novagen) by the removal of Thioredoxin tag, S tag, Nco I and EcoR V restriction sites. The recombinant protein with a hexa-His tag (MHHHHHHSSGLVPRGS) at the N-terminus was expressed in *E*.*coli* BL21 (DE3) in LB medium. The cells were grown in 1 L medium with 100 µg/mL of ampicillin at 37 °C. When the OD_600_ reached ~0.5, isopropyl β-D-1-thiogalactopyranoside (IPTG) was added to a final concentration of 0.1 mM to induce silk protein expression for 12 hours at 20 °C. The cells were re-suspended in 20 mM Tris buffer (pH 8.0, 300 NaCl and 10 mM imidazole) and sonicated for 30 min at 4 °C. The recombinant protein was purified with nickel-nitrilotriacetic acid resin (Qiagen) under native condition according to the manufacturer′s instructions. Imidazole in the eluted protein solution was removed by dialysis against a PBS buffer at 4 °C. Finally, the purified protein solution was stored at 4 °C without agitation.

### Dot blotting analysis

Five BALB/c mice (~20 g) were used for antibody production at the Animal Holding Unit (AHU), Ningxia Medical University. Briefly, purified recombinant TuSp2-RP protein was mixed with Freund’s complete adjuvant (Sigma) at a 1:1 ratio (v/v) to form an emulsion. Each mouse was injected intraperitoneally (i.p.) with 100 μg of recombinant protein. Two booster injections with Freund’s incomplete adjuvant were similarly administered each two weeks later. The animals were bled seven days after the last injection and the serum prepared routinely. Equal amount of dragline silk, eggcase silk, total silks without egg cases and B6 recombinant protein was dot blotted to PVDF membranes (Bio-Rad). Ponceau (0.2%) staining was applied simultaneously to compare the relative amount of the samples. The membranes were blocked for 1 h at room temperature with 5% milk. The primary antibody was anti-sera against recombinant protein (1:200 dilution). The negative control was serum from uninjected normal rats (1:200 dilution). Secondary antibody was HRP-conjugated anti mouse whole IgG (Proteintech). The signals were recorded by the ChemiDoc Touching Imaging System (BioRad).

### Silk materials formation

Recombinant RP was concentrated to ~10 mg/ml at 4 °C by using an Amicon^®^ Ultra Centrifugal Filter (Millipore) according to the manufacturer’s instructions. The concentrated RP solution was stored at 4 °C without agitation. Macroscopic fibers were formed from 250 μL of concentrated RP solution in a 1.5 or 2.0 mL tube upon physical shearing on a bench-top orbital shaker (50–70 rpm) at room temperature. Protein gel can be formed by adjusting pH of as little as 50 μL of concentrated RP solution to ~5.0 at room temperature and film was developed by casting ~100 μL of concentrated RP solution on a petri dish at room temperature.

## Electronic supplementary material

Supplementary Information
