# Supplementary material for: From EST to novel spider silk gene identification for production of spidroin-based biomaterials
Source: Sci Rep. 2017 Oct 17;7:13354. doi: 10.1038/s41598-017-13876-1 (PMC5645381; doi:10.1038/s41598-017-13876-1)
Supplement: Supplementary file 1 — Supplementary Information [file 41598_2017_13876_MOESM1_ESM.pdf]

Supplementary data

From EST to novel spider silk gene identification for production of spidroin-based biomaterials

Weidong Huang[1] , Yan Zhang[1] , Yifan Chen[1], Yin Wang[1], Wensu Yuan[2], Ning Zhang[2], Toong Jin Lam[3], Zhiyuan Gong[3], Daiwen Yang[3*], Zhi Lin[2]*

[1]Department of Biochemistry and Molecular Biology, School of Basic Medicine, Ningxia Medical University, Yinchuan, Ningxia, P.R.China, 750004

[2]School of Life Sciences, Tianjin University, Tianjin, 300072, P.R. China

[3]Department of Biological Sciences, National University of Singapore, 117543, Singapore

Table S1. (part 1): Most abundant cDNA clones—clones with functional relation to translational machinery

cDNA clones	*Frequency*
12 S rRNA gene	16
16 S rRNA gene	64
Elongation factor 1 alpha (domestic silkworm)[a]	15
Elongation factor-2 (*Scolopendra polymorpha*)	11
Elongation factor 1 beta (frog)	7
Translation elongation factor-1 gamma (migratory locust)	6
Translational eukaryotic inititation factor 4AII (EIF4AII) (chicken)	6
Eukaryotic translation initiation factor 5A2 (human)	4
Putative eukaryotic peptide chain release factor subunit 1 (*Polyandrocarpa misakiensis*)	3

Table S1. (part 2): Most abundant cDNA clones—general

cDNA clones	*Frequency*
Ornithine decarboxylase (house fly)	70
ADP/ATP translocase (*Rana rugosa*)	10
Glyceraldehyde-3-phosphate dehydrogenase (gapdh gene) (*Daphnia pulex*)	5
Techylectin-5A (Horseshoe crab)	5
Actin (*Saccoglossus kowalevskii*)	4
Protein transport protein SEC61 alpha subunit (frog)	4
Spidroin 1 (*Nephila clavipes*)	4
Cytochrome oxidase subunit I (COI) gene (*N.a.*)	4
Aldehyde dehydrogenase (RALDH2) (frog)	3
Carboxylesterase precursor (cotton aphid)	3
excretory/secretory mucin MUC-5 (muc-5)(*Toxocara canis*)	3
IgE-dependent histamine release factor (*Dermacentor variabilis*)	3
Ubiquitin gene (human)	3

Table S1. (part 3): Most abundant cDNA clones—heat shock proteins

cDNA clones	*Frequency*
Small heat shock/alpha-crystallin protein precursor (p26-3-6-3) (*Artemia franciscana*)	3
Bombyx mori heat shock protein hsp20.8A	2
Heat shock 70kD protein 5 (human)	1
Hsp70 (African green monkey)	1
Similar to heat shock 60kDa protein 1 (chaperonin) (frog)	1
23 kDa heat shock protein (HSP23) pseudogene (flesh fly)	1

a. The most similar homolog is shown in the brackets.

```
   1  {E  F  A  K  C  M  Y  E  L  V  L  K  S  A  N  S  L  G  V  L
   1 agagtttgcgaagtgtatgtatgaacttgtgcttaaatcagcaaactccttgggcgtact
  21  N  P  H  L  I  A  N  N  I  Y  Q  S  I  V  S  N  L  D  I  L
  61 taaccctcatcttatagccaacaacatatatcaatctattgtgagcaatttggacattct
  41  H  S  S  V  M  I  N  L  Y  A  N  A  M  A  R  N  L  F  H  E
 121 gcactcttcagtgatgataaacctctacgctaacgcgatggccagaaatctttttcatga
  61  G  F  L  N  L  D  N  A  A  T  L  A  K  K  C  A  N  D  M  E
 181 aggcttccttaatttagacaatgccgccacgctcgctaagaaatgcgccaatgacatgga
  81  A  F  A  K  K  M  V  E  T  G}  P  N  L  S  I  G  D  T  T  S
 241 agcctttgccaagaagatggttgaaacaggaccaaacttgtctattggagatactacttc
 101  I  I  Q  L  F  K  N  F  T  G  P  P  S  V  A  T  F  I  S  N
 301 catcatccaattattcaaaaatttacaggtcctccttccgttgcaacattcatctccaa
 121  F  H  S  I  V  Q  S  S  K  T  L  L  N  L  F  D  V  A  E  E
 361 cttccatagcattgtgcaatcttctaagaccttattaaacctctttgacgttgctgagga
 141  N  P  L {E  F  A  K  C  M  Y  E  L  V  L  K  S  A  N  S  L
 421 aaatcctttagagtttgcgaagtgtatgtatgaattggtgcttaaatcagcaaactcctt
 161  G  V  L  N  P  H  L  I  A  N  N  I  Y  Q  S  V  V  S  N  L
 481 gggcgtacttaatcctcatcttattgccaacaacatatatcaatctgttgtgagcaattt
 181  D  I  L  H  S  S  A  M  V  N  L  Y  A  N  A  M  A  G  S  L
 541 ggacattctacattcttcagcgatggtaaacctctacgctaacgcgatggccggaagtct
 201  F  L  E  G  I  L  N  S  D  N  A  A  T  L  A  K  K  C  A  N
 601 atttcttgaaggcatcctcaattcagataatgccgccacgctcgctaagaaatgcgccaa
 221  D  M  E  A  F  A  K  K  M  V  E  I  G}  N  S  I  S  N  I  Q
 661 cgacatggaagcctttgccaagaagatggttgaaataggtaactcgatttcaaatatcca
 241  D  F  P  D  V  S  A  R  I  L  G  N  L  S  L  P  L  L  D  D
 721 agactttcctgatgtatccgcgagaattcttggtaaccttagtctgcccttactggatga
 261  V  L  D  L  L  S  S  F  L  P  *
 781 tgttctcgatcttttgtcttcttttctaccttaattgggttgaactctttcaaaaaagtt
 281
 841 acatttttgaaaatgcatgaaataaattggaatttttggattacacaaaagttcagttat
 301
 901 catatttatagcaacttttataatattttaacttcattccctcctggttgtagtaaagaa
 321
 961 ttttgtgtttttaaattgttttatatatcttgggccatttgtcttcaatatttaaatctt
 341
1021 ccgttcttttagccatttttaagtgaagaatatagactgattctttttgtaatttccaat
 361
1081 aattattttaaatctgcacttgtatttgttgtaatgcaaagaaataagtccttaaaaat
 381
1141 tcgagaatgaataaaaaaacttatttttgaaaaaaaaaaaaaaaaaaaaaaaa
```

Figure S1 Nucleotide and deduced amino acid sequences of the cDNA of EST clone B6. The nucleotide sequence of the clone B6 cDNA (lower line) was obtained from overlapping cDNA fragments and is presented with its deduced amino acid sequence (upper line). Nucleotides are numbered in the 5’ → 3’ direction, whereas amino acids are numbered from the N terminus to the C terminus. Symbol “{ }” and red color are used to indicate the two internal repetitive sequences existing in the cDNA B6. Symbol * is used to indicate the stop codon. Poly A tail are underlined.

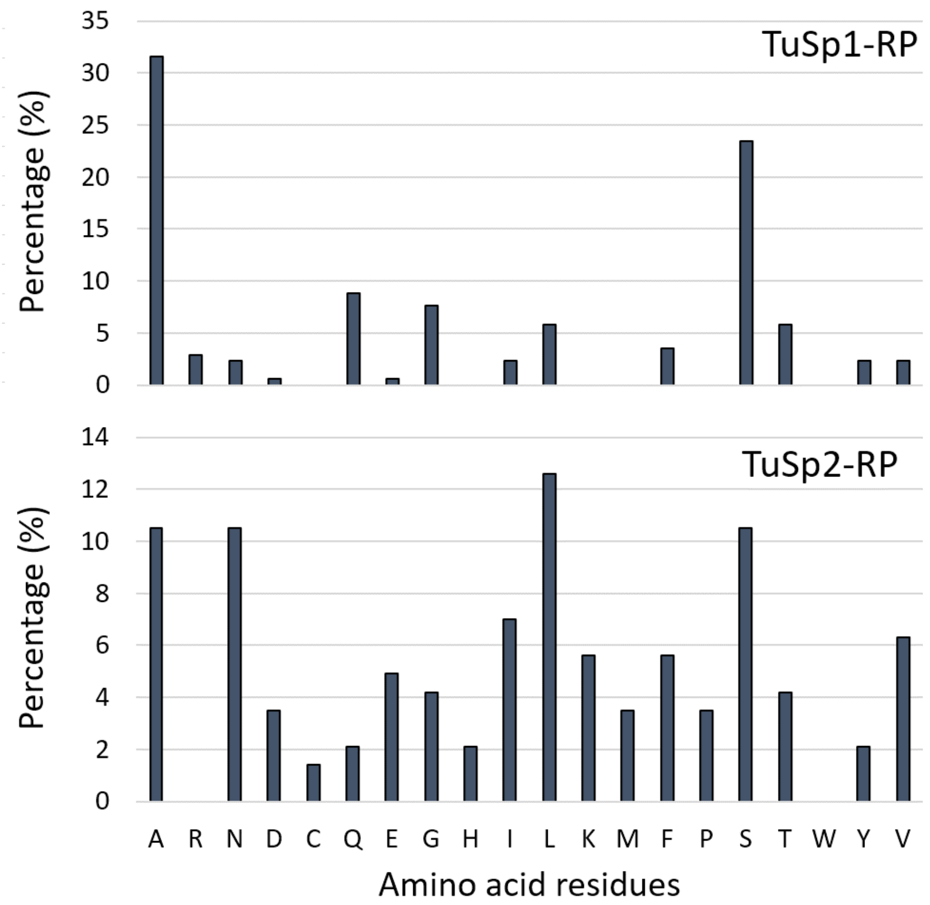

Fig. S2. Amino acid composition of TuSp1 and TuSp2 in RP domain.

```
TuSp1 SSSTTTTTSGATSQAASQSASSSYSSAFAQAASSSLATSSAISRAFASVSSASAASSLAY
MiSp1 -------------------------NAFAQSLSSNLLSSGDFVQMISSTTSTDQAVSVAT
AcSp1 -----------------------LGDQLTSTLASALTKTNTLKAVSASKPSANVAVAIVT
MaSp1 ---------------------------GTRGGQGAGRGGYGGQGAGAGAAAAAAAGGAGQ
TuSp2 ------------------PNLSIGDTTSIIQLFKNFTGPPSVATFISNFHSIVQSSKTLL
                                                    :.  :   :

TuSp1 NIGLSAARSLGIAS--DTALAGALAQAVGGVG-AGASASAYANAIARAAGQFLATQGVLN
MiSp1 SVAQNVGNQLGLDANAMNSLLGAVSGYVSTLGNAISDASAYANAISSAIGNVLANSGSIS
AcSp1 SGLKKALGALRINAGVSSQLTSAVSQAVANVR-PGSSPAVYAKAIAAPSVQILVSSGSVN
MaSp1 GGYGGLGGQGAGQGGLGGQRAGAAAAAAGGAG-----QGGYGGLGSQGAGRGGYGGVGSG
TuSp2 NLFDVAEENPLEFAKCMYELVLKSANSLGVLNPHLIANNIYQSVVSN--LDILHSSAMVN
      .            .          :   .           *    :             .

TuSp1 AVNASSLGSALANALSDSAANSAVSG----------------------------------
MiSp1 ESTASSAASSAASSVTTTLTSYGPAVFY-APTSSAGGYGGLVGY----------------
AcSp1 NNNAKQVASTLSENLVREMANTARRYRVNVPEASVQADVSLVTSMTSTFVISSQTSVQMG
MaSp1 ASAASAAA----------------------------------------------------
TuSp2 LYANAMAGSLFLEGILNSDNAATLAKKCANDMEAFAKKMVEIG-----------------
             .
```

Fig. S3. Sequence alignment among RP domains of TuSp1, MiSp1, AcSp1, MaSp1 and TuSp2. The overall sequence identity among these five sequences ranges from 10-24%.

A

```
N.A.TuSp2  PNLSIGDTTSIIQLFKNFTGPPSVATFISNFHSIVQSSKTLLNLFDVAEENPLEFAKCMY
           P+L IGD TSI QLFK F  PP ++TF+S FH+ VQSS+TLL+LFD+ E  P EF K +
Sbjct      PDLPIGDATSIFQLFKPFPVPPPISTFVSTFHNTVQSSETLLDLFDLTEIKPSEFGKSIG

N.A.TuSp2  ELVLKSANSLGVLNPHLIANNIYQSVVSNLDILHSSAMVNLYANAMAGSLFLEGILNSDN
           E V KSANSLGVLNPH++ANNI  S+ +NLDILHSS M+NLYANA++ ++F EG+LNSDN
Sbjct      EFVQKSANSLGVLNPHIVANNISHSIGNNLDILHSSMMINLYANALSRNMFAEGVLNSDN

N.A.TuSp2  AATLAKKCANDMEAFAKKMVEIGNSISNIQDFPDVSARI
           AA+LAK+ AN+ME  AKK+V   N +S    FP    +I
Sbjct      AASLAKEYANEMEDLAKKIVVKDNPLSKTLVFPAAYEKI
```

B

<u>PDLPIGDATSIFQLFKPFPVPPPISTFVSTFHNTVQSSETLLDLFDLTEIKPSEFGKSIGEF VQKSANSLGVLNPHIVANNISHSIGNNLDILHSSMMINLYANALSRNMFAEGVLNSD NAASLAKEYANEMEDLAKKIVVKD</u>NPLSKTLVFPAAYEKILSKVGLLSLGHVPSVLH HFVNELNLVGELIRKSSTVQNV

Fig. S4 BLAST sequence alignment of TuSp2 from *N. a.* and putative TuSp2 from *N. c.*. **A**. BLAST was performed against the genome of *N. c.* using TuSp2 RP with C-terminal sequence. Sequence identity is ~60% in 159 amino acid residues and E value is $9e^{-53}$. TuSp2 RP domain is underlined. Sbjct: putative TuSp2 from *N. c.*. **B**. Partial sequence of putative TuSp2 from *N.C.*. Putative RP domain is underlined.

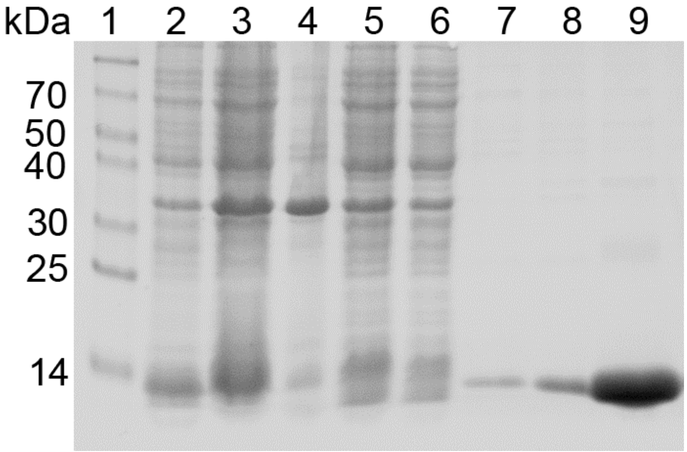

Fig. S5 Purification of recombinant TuSp2-RP by Ni-NTA. Lane 1: protein marker; Lane 2: whole cell lysate, Lane 3: supernatant; Lane 4: pellet, Lane 5: flow-through; Lane 6: wash; Lane7: wash; Lane8-9: elution. Purity of TuSp2-RP is estimated to be ~90%.
